# Spotlight on nurses' smoking prevalence and addiction in Istanbul, Türkiye, the leading country in the implementation of WHO MPOWER policies

**DOI:** 10.1186/s12912-024-02166-7

**Published:** 2024-07-24

**Authors:** Osman Faruk Bayramlar, Gulgun Sabire Uysal, Elif Nur Kocak, Serkan Surme, Selma Karabey

**Affiliations:** 1 Department of Public Health, Istanbul Medical Faculty, Istanbul University, Istanbul, Türkiye; 2 Istanbul Medical Faculty, Directorate of Nursing Services, Istanbul University, Istanbul, Türkiye; 3 Department of Public Health, Istanbul Health Directorate, Sultangazi District Health Directorate, Istanbul, Türkiye; 4 Department of Medical Microbiology, Institute of Graduate Studies, Istanbul University-Cerrahpasa, Istanbul, Türkiye

**Keywords:** Nurses, Prevalence, Smoking, Tobacco, Türkiye

## Abstract

**Objective:**

Türkiye is the leading country that has been applying the MPOWER criteria of the World Health Organization for years. However, the prevalence of smoking among nurses appears to be high, according to the literature. Therefore, we aimed to determine the prevalence, addiction levels, and dynamics of tobacco smoking among nurses in Türkiye.

**Method:**

In this descriptive cross-sectional study, a questionnaire (prepared in cooperation with the “World Health Organization”) and the Fagerström Test for Nicotine Dependence were administered to 529 nurses working at a tertiary-care university hospital in 2020. Logistic regression was performed to determine factors predicting smoking.

**Results:**

The prevalence of smoking among nurses was 32.7% (*n* = 173). The mean Fagerström test score indicated a "low dependence" level (score: 3 ± 2.6). Both results were higher for males. A relationship was found between trying smoking cigarette and hookah. Of the “current smokers” group, 102 (60.4%) stated that they wanted to quit smoking. Only 21 (27.6%) of the nurses who have tried to quit smoking thus far have received professional help.

**Conclusion:**

The prevalence of smoking among nurses working at a tertiary-care university hospital was relatively low compared to that among nurses in Türkiye. While females are normally expected to smoke less, the high prevalence of smoking among nurses (most of them female) raises the question of the professional basis of this situation. However, the low rate of receiving professional help reveals the lack of promotion and accessibility of smoking cessation outpatient clinics in the faculty environment. Finally, the perception that hookah is an alternative tobacco product leads to cigarette smoking. The good news was that nurses had a low dependency rate.

**Supplementary Information:**

The online version contains supplementary material available at 10.1186/s12912-024-02166-7.

## Introduction

Five years after the World Health Organization (WHO) accepted the Framework Convention on Tobacco Control in 2003, it announced and reported the MPOWER criteria, which point to six basic practices in tobacco control. Türkiye has been a leading country that has implemented all of these criteria in reports since 2013 and is cited as an example [1, 2].

In the fight against tobacco, it is very important to help smokers and their families quit smoking, and healthcare professionals have important duties in this regard [3–5]. A meta-analysis revealed that interventions by nurses in this role are more likely to help them quit smoking than interventions by other people [6, 7].

However, it would be unrealistic to expect smoking control interventions from nurses who smoke themselves [8]. This is because smoking nurses will likely be less motivated to offer these interventions and have a less positive attitude toward smoking cessation [9]. Studies in the literature have shown that health workers consume more cigarettes than does the general public. Moreover, many studies have shown that nurses consume more cigarettes than other healthcare workers [10, 11]. Therefore, targeting the nurse profession in the fight against smoking would be a correct target, as they are a group of smokers at a high rate and have a high potential to help others quit smoking.

For the correct targeted intervention, we must address and understand the problem comprehensively and systematically. There are scales in the literature to systematically monitor tobacco use and control indicators among adults, and the Global Adult Tobacco Survey (GATS) is a prominent example of this [12]. While the GATS was being created, it was ultimately approved by a sizable committee and customized to each nation's language [13]. Türkiye conducted GATS in 2008, 2012, and 2016 as one of the first nations in the world to do so [14–16]. As a result, tools that use this scale can provide us with accurate comparisons.

The nicotine in tobacco products is addictive. Various scales around the world measure this phenomenon. A widely known scale called the Fagerstrom Test for Nicotine Dependence was developed in 1978 [17]. The extent of dependency may indicate how realistic a potential intervention would be.

Considering these arguments and tools, we aimed to determine the prevalence of tobacco use behavior, addiction levels, and dynamics and their relationships with various sociodemographic factors to formulate effective tobacco control measures for nurses.

## Materials and methods

### Study design, population, sample, and power analysis

Between August and November 2020, information was gathered from nurses working at Istanbul University, Istanbul Medical Faculty, the first medical school in Türkiye, which has been educating people for more than five centuries [18]. During this period, there were 896 nurse employees.

**Fig. 1:**
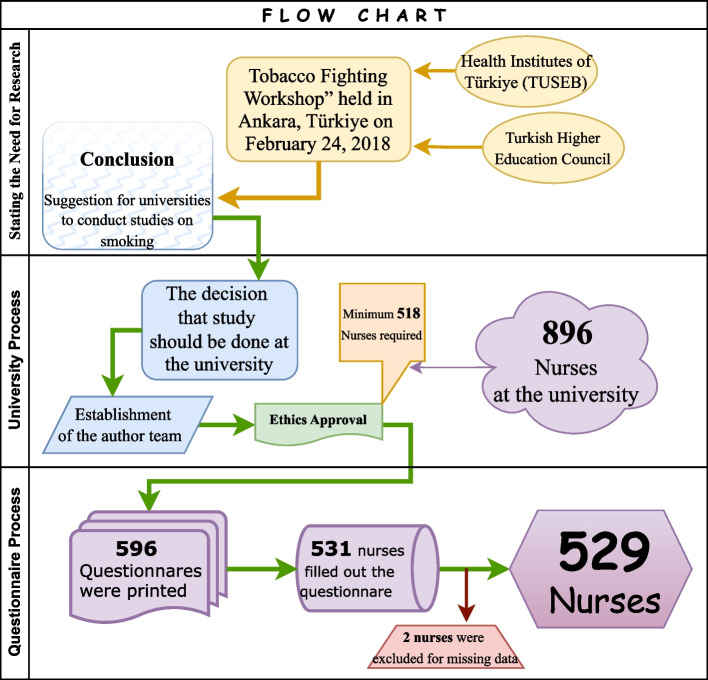
Flow Chart

In this descriptive cross-sectional study, we performed a power analysis to determine the minimum sample size required for the study. We accepted the effect size as 0.4, which is known as a moderate level. In addition, type I error was taken as the 5% level, and the power level was accepted as 80%. Accordingly, the sample size to be reached was calculated to be 518. A further 15% of the required sample size was added to account for potential losses during the study and the possibility that individuals would withdraw for various reasons. Therefore, we aimed to collect questionnaires from 596 nurses. The response rate to the questionnaire was 89.1%. The sample was selected using the nonprobability convenience sampling method. Nursing students were not included in the study. Nurses who did not give complete informed consent or complete a questionnaire were excluded from the study (Fig. 1).

In addition, in order to see the trend in Türkiye and analyze it correctly, we tabulated and mapped the studies conducted in the last 15 years on nurses' smoking histories, scanned in PubMed, Google Scholar and Higher Education Council Thesis System (YOKTEZ), in the discussion section. We found 46 studies on this topic (Supplementary Table 1). The last date we collected data was June 20, 2023.

### Questionnaire

The Turkish questionnaire, consisting of 66 multiple-choice questions, was prepared by a consortium comprising the Turkish Statistical Institute (TURKSTAT), the Ministry of Health of the Republic of Türkiye, the Ministry of National Education of the Republic of Türkiye, and the WHO Türkiye Office. The questions were derived from the English versions of the 'Tobacco Questions for Surveys (TQS) [19]', 'Global Adult Tobacco Survey (GATS) [16]', and 'Global Youth Tobacco Survey (GYTS) [20]', developed in collaboration with the WHO and the Centers for Disease Control and Prevention (CDC). The finalized questionnaire was provided to us by Prof. Toker Erguder, the program manager of the WHO Türkiye Office, and has been previously utilized and published in our prior tobacco research [21]. The 66 questions are categorized as follows: Tobacco Use Frequency (25 questions), Environmental Tobacco Exposure (4 questions), Attitudes (12 questions), Behavior/Tobacco Cessation (10 questions), Curriculum/Education (8 questions), and Socio-Demographic Features (7 questions).

The nurses' sociodemographic and socioeconomic traits, tobacco use histories, quitting experiences or opinions, status as passive smokers, knowledge and attitudes toward tobacco use, and tobacco policies were all examined.

### Fagerström Test for Nicotine Dependence (FTND)

**Fig. 2:**
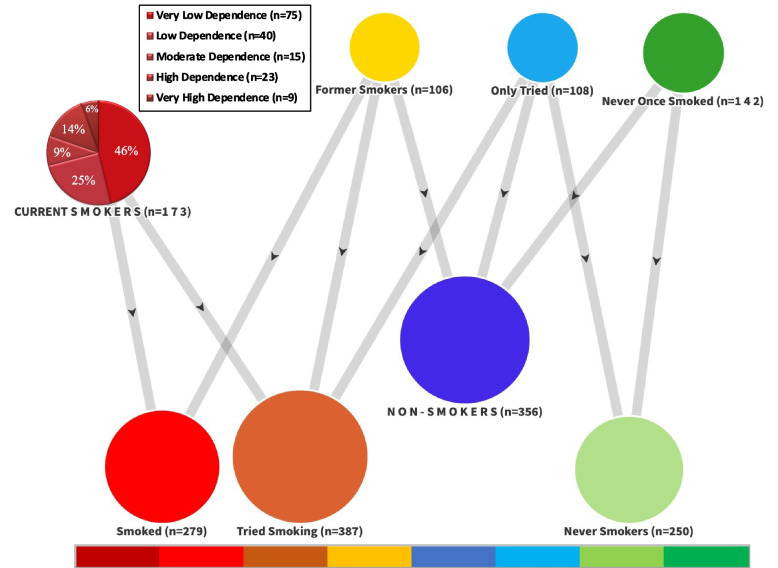
Display of 8 Smoking Statuses, 3 of Which are Categorized, Produced from 4 Main Data

The Fagerström Tolerance Questionnaire (FTQ) was developed by Fagerström in 1978 to assess nicotine dependence [17]. In 1991, Heatherton et al. developed the FTND, a shorter questionnaire version with higher internal consistency [22]. The FTND is a 6-item scale. Uysal et al. assessed the validity of the test in the Turkish population [23]. The maximum score that can be obtained is 10. In contrast, the lowest possible score is 0. The cutoff points were determined to be 0–2 points for very low dependence, 3–4 points for low dependence, 5 points for moderate dependence, 6–7 points for high dependence, and 8–10 points for very high dependence [24] (Fig. 2).

### Dependent variables

The five cutoff points for FTND were also dichotomously categorized as "very low dependence" or "low dependence".

According to the Centers for Disease Control and Prevention (CDC), since 2004, there have been 4 groups known as SMKSTAT2 codes and defined by smoking status: "*Current Every Day* (previously called Regular) *Smokers*", "*Current Someday* (previously called Occasional) *Smokers*", "*Former Smokers,*" and "*Never Smokers*" [25].

In this research questionnaire, we assessed smoking status with four primary groups: "*Current Smokers*", "*Former Smokers*", "*Only Tried*" and "*Never Once Smoked*".

- *Current Smokers***:** Smoking status defined in SMKSTAT1, which is the combination of 2 groups in SMKSTAT2 ("Current Every Day" + "Current Smokers").
- *Former Smokers*: It is defined in SMKSTAT2.
- Never Smokers: We collected this SMKSTAT2 definition in more detail and divided it into two parts:
  - Only Tried: People who have smoked or tried 1-100 cigarettes in their lifetime.
  - Never Once Smoked: People who have never smoked even once.
- Smoked: Groups where "Current Smokers" and "Former Smokers" are combined and evaluated.
- Tried Smoking: A group was formed to compare the attitudes of people who had smoked at least once with those of people who had not smoked.
- Non-Smokers: A group was created to compare smokers with a control group.

### Statistical analysis

Continuous quantitative data are expressed as the mean and standard deviation. Categorical data are presented as percentages (%) and frequencies (n). The Pearson chi-squared test was used to compare qualitative characteristics. For continuous data analysis, the Kolmogorov‒Smirnov test, box plot, mean‒median measures, and kurtosis–skewness values were used to assess the normality of the distribution of the data for parametric test selection. The Kruskal–Wallis test was used to analyze continuous and more than two independent nonparametric groups, and the Dunn–Bonferroni correction was used for post hoc analysis. Univariate and multivariate logistic regression were used for the data that were found to be significant (the dependent variables were "Current Smoker//Nonsmoker" and "Very Low//Low Dependence" as binary variables). In the tests, which of the significant independent variables differed was examined by calculating the effect size (η^2^, R^2^). The results were evaluated with a 95% confidence interval, and the statistical significance level was set at *p* < 0.05. The analyses were performed using IBM SPSS-21 (Statistical Package for Social Sciences, Chicago, IL, USA).

## Results

A total of 529 nurses were included in this research. Of those, 483 (91.3%) were female (Table 1).

**Table 1:** Smoking Status of Nurses by Sociodemographic and Socioeconomic Data

FACTORS			CURRENT SMOKERS*32.7% (n=173)*		NON-SMOKERS*67.3% (n=356)*		N	%*	**p** **η**^2^
			**n**	%	**n**	%			
Sex	**Female [♀]**		147	*30.4*	336	*69.6*	483	91.3%	**001** **η**^2^:.025
	**Male [♂]**		26	*56.5*	20	*43.5*	46	8.7%	
Age	**19-29**		40	*29.9*	94	*70.1*	134	25.3%	.39
	≥ **30**		123	*33.7*	262	*66.3*	395	74.7%	
Income level	≤**Medium**		155	*31.8*	332	*68.2*	487	92.1%	.14
	>**Medium**		18	*42.9*	24	*57.1*	42	7.9%	
Marry	**Single**		82	*35.3*	150	*64.7*	232	43.9%	.21
	**Married**		91	*30.6*	256	*69.4*	297	56.1%	
Being Orphan	**No**		151	*32.3*	316	*67.7*	467	90.2%	.89
	**Yes**		17	*33.3*	34	*66.7*	51	9.8%	
Having Partner	**No**		26	*33.8*	51	*66.2*	77	14.9%	.93
	**Yes**		145	*32.9*	296	*67.1*	441	85.1%	
**Partner**	**SOCIAL CIRCLE SMOKING**	**No**	57	*22.9*	192	*77.1*	249	56.5%	**.001** **η**^2^:.059
		**Yes**	88	*45.8*	104	*54.2*	192	43.5%	
**Mother**		**No**	141	*31.3*	310	*68.7*	451	86.2%	**.05** **η**^2^:.007
		**Yes**	31	*43.1*	41	*56.9*	72	13.8%	
**Father**		**No**	102	*28.7*	253	*71.3*	355	67.9%	**.003** **η**^2^:.017
		**Yes**	70	*41.7*	98	*58.3*	168	32.1%	
**Parent**		**No**	76	*26.8*	208	*73.2*	284	60.2%	**.001** **η**^2^:.025
		**Yes**	79	*42*	109	*58*	188	39.8%	
**Best Friend**		**No**	36	*16.4*	184	*85.6*	220	42.3%	**.001** **η**^2^:.091
		**Yes**	135	*45.1*	165	*54.9*	300	57.7%	

*Column percentage

### Smoking status

One hundred seventy-three nurses (32.7%) were "current smokers", 106 (20%) were "former smokers", 108 (20.4%) were in the "only tried" group, and 142 (26.8%) had "never once smoked" (Fig. 2). Thirty-nine (19.7%) of the current smokers had smoked for five years or more.

Smoking was more common in males than in females (56.5% vs. 30.4%, *p* < 0.001). Age, income level, marital status, and partner presence did not affect smoking status. However, we found that social circle smoking significantly affected nurses' smoking status (*p* < 0.05).

### Nicotine dependence status

The mean Fagerström test score in current smokers was 3 ± 2.6. This score is accepted at the "low dependence" level. It was found to be significantly greater in males than in females. (4.5 vs. 2.7, *p* = 0.003). Smoking years was another critical factor, and the test score was found to be significantly greater for those who had smoked for 5 years or more (3.2 vs. 2.1, *p* = 0.04). In addition, the mean score was significantly greater for those whose parents died (4.4 vs. 2.8, *p* = 0.01) and those whose fathers smoked (3.6 vs. 2.4, *p* = 0.02) (Table 2).

**Table 2:** Fagerström Test Scores of Current Smokers by Sociodemographic and Socioeconomic Data

**FAGERSTRÖM TEST**			**N**	**Mean**	**SD**	**Median**	**25per**	**75per**	**p** **η**^2^
**Current Smokers**			162	3	*2.6*	3	1	5	
**Sex**	**Female (♀)**		138	2.7	*2.5*	3	0	5	**.003** **η**^2^:.062
	**Male (♂)**		24	4.5	*2.9*	4.5	2	6.5	
Age	**19-29**		40	2.9	*2.4*	3	1	4.5	.89
	≥** 30**		122	3	*2.7*	3	0	5	
Income level	**≤Medium**		144	2.9	*2.6*	3	0	5	.16
	**>Medium**		18	3.8	*2.7*	3	2	6	
**Smoking Years**	**0-4 years**		29	2.1	2.2	2	0	3	**.04** **η**^2^:.027
	**³ 5 years**		133	3.2	2.7	3	1	5	
Want to Quit	**No**		63	3.3	2.7	3	1	6	.16
	**Yes**		96	2.8	2.6	2.5	0	5	
Professional Help	**No**		119	2.8	2.7	3	0	5	.1
	**Yes**		39	3.5	2.4	4	2	5	
Marry	**Single**		77	3.4	*2.8*	3	1	5	.1
	**Married**		85	2.6	*2.4*	2	0	4	
**Being Orphan**	**No**		142	2.8	2.6	3	0	5	**.01** **η**^2^:.033
	**Yes**		16	4.4	2.1	5	3	6	
Having Partner	**No**		23	3.7	*2.6*	3	1	6	.12
	**Yes**		139	2.9	*2.6*	3	0	5	
Partner	**SOCIAL CIRCLE SMOKING**	**No**	55	3.1	*2.9*	2	1	5	.69
		**Yes**	84	2.7	*2.4*	3	0	5	
Mother		**No**	131	3.6	3.1	3	0	6	.21
		**Yes**	31	2.8	2.5	3	1	5	
**Father**		**No**	94	2.6	2.3	2	1	4	**.05** **η**^2^:.037
		**Yes**	68	3.6	2.9	3	1	6	
**Parent**		**No**	80	2.5	2.3	2	1	4	**.05** **η**^2^:.036
		**Yes**	82	3.4	2.9	3	1	6	
Best Friend		**No**	35	2.9	*2.7*	3	1	5	.73
		**Yes**	126	3	*2.6*	3	1	5	

When these Fagerström test scores are classified according to 5 cutoff points, we see that "very low dependence" (*n* = 75, 46.3%) is the highest (Fig. 2).

### Reasons to start smoking

Thirty-five (20.2%) of the "current smokers" tried their first cigarette under the age of 16, while 101 (58.4%) tried under the age of 18.

When the reasons for starting smoking were examined in the "smoked" group, "having smoker best friends" was the factor with the highest rank for 123 people (34.9%). Other factors were "stress" for 62 people (17.6%) and "curiosity" for 59 people (16.8%). In addition to these factors, factors such as "emulation", "personal reasons", "family factors", "feeling of freedom", and "desire to show that one has grown" were also identified.

### The situations in which the highest cigarette consumed

Of the "smoked" group, 69 people (39.9%) smoked more during stressful times; 49 people (28.3%) also stated that they consumed more cigarettes while together with their friends.

### Efforts to quit smoking

In the "current smoker" group, 102 people (60,4%) stated that they wanted to quit smoking. In addition, although the Fagerström test score of those who wanted to quit smoking was not significantly different, it was lower than that of those who did not want to quit smoking (2.8 vs. 3.3, *p* = 0.16) (Table 2).

Seventy-seven (27%) of the "smoked" group stated that they had tried to quit smoking within the last year. When the reasons for their desire to quit were examined, "health problems" was the most effective variable, with 26 (41.9%). Another important factor was "family's factor", accounting for 7 (11.3%) respondents. Apart from this, "cigarette's price" and "friend's factor" were effective.

Of 77 nurses who tried to quit smoking, 21 (27.6%) received professional help at least once.

### The relationship between trying hookahs and cigarette smoking

There was a direct proportional relationship between knowing that "hookah is tobacco" and trying hookah (*p* = 0.02, OR = 1.93). Nurses who tried to use hookah were more likely to be in the 'tried smoking' group than those who did not (*p* < 0.001, OR: 5.32). Current smokers were more likely to try hookah than nonsmokers were (*p* < 0.001, OR: 3.33).

### The relationship between healthcare workers' possible attitudes toward smokers and their smoking status

Four hundred and four (77.7%) of the nurses stated that healthcare workers should advise their patients to quit smoking on a regular basis, and 344 (68%) stated that this would be effective. Those who opposed this statement were more likely to be current smokers (OR: 2.04, *p* < 0.001).

### Multivariate regression analysis of smoking causes

Multivariate regression analysis revealed that "male sex", "best friend's smoking", "partner's smoking", and "parent's smoking" were the most influential factors. These factors could explain 23.5% (Nagelkerke R Square) of the variance in smoking (Table 3).

**Table 3:** Multivariate Regressions of the Factors Most Influencing Smoking and Nicotine Dependency

**SMOKING**	**Univariate**				**Multivariate**			
	**Odds Ratio**	**95% CI**		**P R**^**2**^	**Odds Ratio**	**95% CI**		**P R**^**2**^
		**Lower**	**Upper**			**Lower**	**Upper**	
**Male [♂]**	2.97	1.61	5.49	** < .001** **R**^**2**^**:**.025	3.17	1.46	6.89	**.004** **R**^**2**^:.171
**Best Friend’s Smoking**	4.18	2.74	6.39	** < .001** **R**^**2**^:.091	3.79	2.26	6.36	** < .001** **R**^**2**^:.171
**Partner’s Smoking**	2.85	1.89	4.29	** < .001** **R**^**2**^:.059	2.63	1.63	4.22	** < .001** **R**^**2**^:.171
**Parent’s Smoking**	1.98	1.34	2.93	** < .001** **R**^**2**^:.025	1.88	1.18	2.99	**.007** **R**^**2**^:.171
**NICOTINE DEPENDENCY**	**Univariate**				**Multivariate**			
**Being Orphan**	4.21	1.15	15.42	**.03** **R**^**2**^:.034	5.05	1.33	19.11	**.02** **R**^**2**^:.072
Male [♂]	2.36	0.92	6.05	.07	2.49	0.94	6.56	.07 **R**^**2**^:.072
Father’s Smoking	1.81	0.94	3.47	.08	1.84	0.94	3.59	.07 **R**^**2**^:.072
Smoking Years	1.83	0.81	4.14	.14				

In another multivariate regression for nicotine dependency, factors such as "being orphaned", "male sex", and "father's smoking" were the most influential predictors. These factors could explain 10% (Nagelkerke R Square) of nicotine dependency (Table 3).

## Discussion

This research examined the attitudes, practices, and dynamics of tobacco smoking among 529 nurses in a tertiary-care university hospital. The prevalence of smoking was 32.7%, and one out of five nurses had a high dependence on nicotine.

**Fig. 3:**
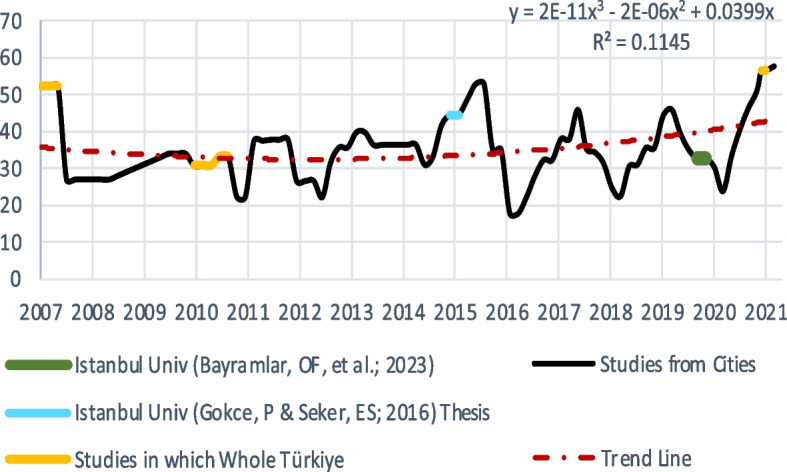
The Prevalence of Smoking Studies Conducted on Nurses in Türkiye since 2007

All prevalence studies published on nurses' smoking in Türkiye in the last 15 years ranged between 17.8% and 57.7% (Supplementary Table 1). When we drew a trend line of this 15-year prevalence and while a downward trend was observed until 2014, a significant upward trend was observed from 2014 to the present (Fig. 3). However, within this trend analysis, we also referenced a single study conducted at our faculty in 2015, which utilized a smaller sample size and reported a prevalence rate of 44.5% (*n* = 200) [26]. Encouragingly, our results indicate that nurses at our faculty have deviated from this trend, reducing the prevalence from 44.5% observed post-2014 to the current rate of 32.7%. Our institution's status as a tertiary-care university hospital, which potentially underscores a heightened commitment to tobacco control efforts, may be responsible for this improvement. Future research encompassing broader, multicenter studies among nurses within tertiary-care university hospitals could further elucidate the factors contributing to this positive trend.

In the last 15 years, four studies on smoking prevalence among nurses across Türkiye as a whole were identified. The most inclusive intervention was the GATS-2012; according to the findings, the percentage of nurses who smoke regularly was only 19.2% [15]. However, occasional smokers were not included in this analysis, but we also included this group in this research. Thus, we searched for studies that also covered occasional smokers. In a news item in the press, we found a statement from the Ministry of Health of the Republic of Türkiye about GATS-2012 that provides additional information. In this statement, the prevalence of smoking among nurses was 33.2% when occasional smokers were included in the GATS-2012 [27]. In the GATS-2008, the prevalence of regular and occasional smokers combined was 55.2% [14] (Supplementary Table 1). In essence, these studies corroborated the declining trend observed up to 2014.

**Fig. 4:**
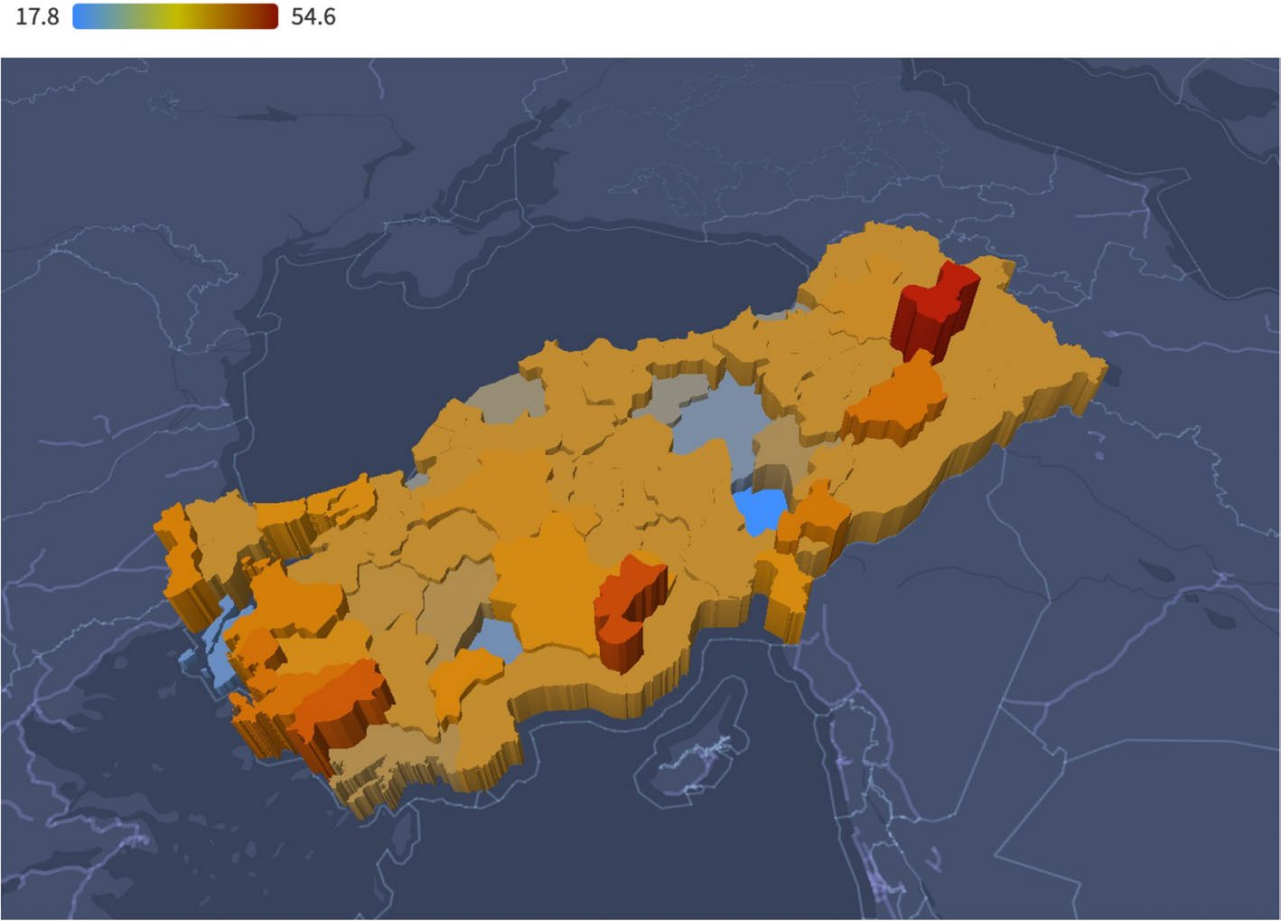
Map of the Mean Course of Smoking among Nurses in Different Cities in Türkiye between 2007 and 2022

Apart from these 4 studies, 42 studies were carried out in 29 different cities (81 cities in total) at different times between 2007 and 2022 (details are given in Supplementary Table 1). Three of these cities, Mus, Karaman, and Aydin, had higher prevalence rates (Fig. 4). Istanbul's prevalence is close to Türkiye's average, and it is the most populous city in the country, indicating that it is a good city choice to generalize Türkiye.

When we look at the general course of smoking among nurses in Türkiye, while the smoking rate was approximately 35% in 2008, it started to decrease to 30% in 2014 and then increased to over 40% in 2021. Türkiye has become one of the leading countries in the world in terms of the WHO's MPOWER criteria, with its tobacco control policies implemented in the early 2000s [1, 28–31]. The smoking-reducing effect of these policies was also observed in our findings. However, although these policies have not returned, the increasing trend observed both in the WHO MPOWER reports and in this research is worrying (Fig. 4). In addition, in the 2021 report, Türkiye was also unsuccessful in e-cigarette mapping [1, 2]. According to one interpretation, this might be because cigarette companies and individual consumers have now developed resistance to some laws [31]. Additionally, the frequency of breaking laws and regulations has significantly increased [29, 32, 33]. We can infer from these results that the implementation of the tobacco control program has not been effective in recent years.

According to Turkstat data, the percentages of male and female individuals who use tobacco daily among individuals older than 15 years in Turkey between 2010 and 2022 are 41.3% and 15.5%, respectively [34]. This research revealed that the smoking rate of males was greater than that of females (56.5% vs. 30.4%). Additionally, Turkstat data include occasional smokers, and 32.1% of the Turkish population are current smokers. This rate is also less than 20% for females [34]. However, when we look at the trend line in Fig. 4 for nurses, who are mostly females, this rate seems to be above 40%. A study emphasized that smoking among females is associated with concepts such as modernity, emancipation, and independence [35]. Having a profession accepted in the community, such as nursing, may also parallel these impulses.

Studies have shown that stress is the primary factor increasing the prevalence of smoking, and high levels of stress are associated with an increased prevalence of smoking, especially among females [36, 37]. This research supports these results. In addition, the demanding working conditions and stress of the nursing profession may explain these high prevalence levels.

The rates of starting smoking in Türkiye were between 15–19.6% before the age of 15 and between 57.5–58.9% before the age of 18 [14–16]. In this research, we found rates very close to these findings. This shows the worsening trend in the smoking epidemic in Türkiye.

According to the literature, the prevalence of nurse smoking in Cyprus is 28.1% [38], that in Greece is 32% [39], that in Bosnia and Herzegovina is 51% [40], that in Italy [41]is 36%, that in Spain and the United States is between 4 and 30% [42], that in Ireland is 21% [43], and that in New Zealand is 8%. This rate decreased from 13.6% in New Zealand after 2006 [44]. While there is a decline in the smoking rates of nurses in high-income countries, the upward trend in Türkiye is thought-provoking [45].

The increase in smoking is the result of various factors. According to a study conducted in 2005, the influence of friends ranks first, with 36.7% of the reasons for starting smoking [46]. A circle of friends was once again cited as the top cause for beginning smoking in a study from 2012 [47]. In this research, smoking was found to be significantly more common among nurses whose best friends, partners, mothers, or fathers were current smokers. The "close friend" category was identified as the top factor in smoking initiation. In the multivariant regression, which explained 23.5% of the reasons for smoking, we found that the most important factor that emerged again was "best friend smoking" (Table 3). This may show that nurses perceive smoking as a way of socializing and that peer interactions should also be considered when determining intervention areas.

There were 12 studies that applied the FNBT to determine smoking prevalence among nurses in Türkiye. The range of the means measured in these studies was between 3 and 4. This score is called "low dependence". According to our categorical analysis, "(very) low dependence" was predominantly inconsistent with this research (Supplementary Table 1). Thus, we believe that smoking cessation success rates would be high if the right interventions were conducted.

A 2013 study in Türkiye found that 62.6% of current smokers considered quitting [48], with a similar rate of 60.4% in this research. However, the percentage of those who tried to quit smoking in the last year was relatively low. A low percentage of these groups received professional help. This reveals the lack of promotion and accessibility of outpatient clinics for smoking cessation activities in the faculty environment.

In this research, nurses who tried hookah had a greater chance of smoking than did those who did not. According to a systematic review, compared with males, females were more prone to hookah use than other tobacco products [49]. When these facts are taken into account along with the results of this research, it becomes clear that these people have a high risk of developing a smoking habit. To further reduce the relatively low rate of tobacco use among females, the use of tobacco products such as hookahs and e-cigarettes as alternatives to quitting smoking should be avoided [50].

The lack of a multicenter, randomized sample and the absence of a prospective study should be considered limitations.

## Conclusion

At a tertiary care university hospital, one-third of nurses smoke actively. This high rate was relatively low compared to that of nurses in Türkiye. While females are normally expected to smoke less, the high prevalence of smoking among nurses (most of whom are female) raises the question of the professional basis of this situation. We are concerned that the nurses, whom we expect to play a leading role in the tobacco fight, have taken a path that contradicts our expectations of the medical faculty. Most smokers are thinking about quitting and have low levels of dependence. Therefore, bringing these nurses together with smoking cessation counseling services may be beneficial. However, the low rate of receiving professional help reveals a lack of promotion and access to smoking cessation outpatient clinics in the faculty environment. Finally, we realized that the perception of hookah as an alternative tobacco product has the potential to induce nurses to start smoking in the future.

### Supplementary Information

Supplementary Material 1.

## Data Availability

The dataset used or analyzed during the current study are available from the corresponding author on reasonable request.

## Code availability

The codes used throughout the study are available from the corresponding author upon reasonable request.
