# Supplementary material for: Spotlight on nurses' smoking prevalence and addiction in Istanbul, Türkiye, the leading country in the implementation of WHO MPOWER policies
Source: BMC Nurs. 2024 Jul 24;23:505. doi: 10.1186/s12912-024-02166-7 (PMC11267963; doi:10.1186/s12912-024-02166-7)
Supplement: Supplementary file 1 — Supplementary Material 1. [file 12912_2024_2166_MOESM1_ESM.docx]

**Supplementary Table 1.** Smoking Prevalence and FNBTs of Nurses between 2007 and 2022 in Türkiye

| **NO** | **Year** | **Placement** | **Reference** | **N** | **%** | **FNBT** | **FNBT Nicotine Dependency** | | | | |
| --- | --- | --- | --- | --- | --- | --- | --- | --- | --- | --- | --- |
|  |  |  |  |  |  | **Mean±SD** | **Very Low** | **Low** | **Mode rate** | **High** | **Very High** |
| **1** | 2007 | TÜRKİYE | GATS-2008 [14] | 690 | 52.3 |  |  |  |  |  |  |
| **2** | 2008 | Trabzon | Muslu, C; et al.; 2012 [51] | 100 | 27 |  |  |  |  |  |  |
| **3** | 2010 | Erzurum | Saglam, L, et al; 2010 (10) | 260 | 34 |  |  |  |  |  |  |
| **4** | 2010 | TÜRKİYE | Sonmez, CI, et al.; 2015 [52] | 1,340 | 30.7 | 3.4 ± 2.2 |  |  |  |  |  |
| **5** | 2011 | TÜRKİYE | GATS-2012 [15] | 690 | 33.2 |  |  |  |  |  |  |
| **6** | 2011 | Ankara | Uz, D & Kitis, Y; 2011 [53] ***** | 538 | 22.3 |  |  |  |  |  |  |
| **7** | 2011 | Ankara | Bilgin, G; et al.; 2012 [54] | 357 | 37.5 |  |  |  |  |  |  |
| **8** | 2012 | Edirne | Serez, B & Karlikaya, HC; 2013 **[55] *** | 380 | 37.8 |  | 68.4 | 18.4 | 5.9 | 5.9 | 1.5 |
| **9** | 2012 | Duzce | Ustun, A & Mayda, AS; 2014 [56] ***** | 154 | 26.6 |  |  |  |  |  |  |
| **10** | 2013 | Tokat | Demir, S & Citil, R, 2016 [57] ***** | 158 | 22.2 |  |  |  |  |  |  |
| **11** | 2013 | Ankara | Karaardic, L & Kir, T; 2013 [45] ***** | 511 | 31.6 |  | 63 | 15.3 | 8.9 | 9.6 | 3.2 |
| **12** | 2013 | Hatay | Aliskin, O, et al.; 2015 [58] | 101 | 35.7 | 3.6 ± 2.8 | 39.3 | 21.4 | 14.3 | 17.9 | 7.1 |
| **13** | 2013 | Gaziantep | Ozturk, M & Aydin, N; 2015 [59] ***** | 196 | 39.8 |  |  |  |  |  |  |
| **14** | 2014 | Burdur | Sahin, DS; et al.; 2014 [60] ***** | 140 | 36.4 |  |  |  |  |  |  |
| **15** | 2015 | Mugla | Mandas, U & Irmak, Z; 2018 [61] ***** | 200 | 31 |  |  |  |  |  |  |
| **16** | 2015 | Izmir | Cakar, S & Ocek, ZA; 2017 [62] ***** | 169 | 33.1 |  |  |  |  |  |  |
| **17** | 2015 | Diyarbakir | Sen, MA & Palanci Y; 2016 [63] ***** | 334 | 41.6 |  |  |  |  |  |  |
| **18** | 2015 | Istanbul | Gokce, P & Seker ES, 2016 [26] ***** | 200 | 44.5 |  |  |  |  |  |  |
| **19** | 2015 | Karaman | Yasar, H & Kara, F; 2019 [64] ***** | 315 | 48.6 |  | 57.5 | | 10.5 | 32 | |
| **20** | 2016 | Mus | Sezgin, L & Pirincci, E; 2020 [65] | 436 | 52.9 |  | 43.9 | 18.7 | 11.7 | 18.7 | 7 |
| **21** | 2016 | Manisa | Ozvurmaz, S & Yavas, S; 2018 [66] | 367 | 35.1 |  |  |  |  |  | 47.1 |
| **22** | 2016 | Kahramanmaras | Alici, OS & Sucakli, MH; 2016 [67] ***** | 45 | 17.8 |  |  |  |  |  |  |
| **23** | 2017 | Istanbul | Uncu, B & Ustundag, FH; 2017 [68] ***** | 293 | 22.5 |  |  |  |  |  |  |
| **24** | 2017 | Kastamonu | Uzer, Fatih; 2018 [69] | 110 | 28.1 | 4.04±2.81 |  |  |  |  |  |
| **25** | 2017 | Kutahya | Ozdogan, N&Kosgeroglu, N; 2018 [70] ***** | 720 | 32.3 |  |  |  |  |  |  |
| **26** | 2017 | Balikesir | Cirik, A & Goksel, A; 2018 [71] ***** | 200 | 38 |  |  |  |  |  |  |
| **27** | 2018 | Aydin | Ozaydin, E & Gezer, N; 2019 [72] ***** | 170 | 45.9 |  |  |  |  |  |  |
| **28** | 2018 | Konya | Marakoglu K & Unal, GU; 2019 [73] | 79 | 35.4 |  | 44.4 | 25.8 | 9.6 | 12.1 | 8.1 |
| **29** | 2018 | Istanbul | Sagiroglu B & Yilmaz, HO; 2019 [74] ***** | 180 | 34.4 |  |  |  |  |  |  |
| **30** | 2018 | Tokat | Kutukcu, E & Kocatas, S; 2019 [75] | 200 | 31.5 | 3.3 ± 2.4 |  | 54 |  |  |  |
| **31** | 2018 | Sivas | Soyak, R & Yilmaz, M; 2019 [76] ***** | 215 | 24.7 |  |  |  |  |  |  |
| **32** | 2018 | Canakkale | Mutlu, P, et al.; 2019 [77] ***** | 71 | 22.5 |  | 5.8 | 21,2 | 46 | 17.5 | 6.6 |
| **33** | 2019 | Afyon | Cilekar, S & Gunay, E; 2020 [78] | 300 | 31 |  |  |  |  |  |  |
| **34** | 2019 | Kocaeli | Esim F & Caliskan, I; 2019 [79] ***** | 132 | 35.6 |  |  |  |  |  |  |
| **35** | 2019 | Izmir | Hassoy, D & Ozvurmaz, S; 2021 [80] | 131 | 44.3 |  | 6.6 | 17.4 | 11.6 | 17.4 | 47.1 |
| **36** | 2019 | Istanbul | Neziroglu, D & Akyolcu, N; 2021 [81] ***** | 289 | 46 |  |  |  |  |  |  |
| **37** | 2020 | Istanbul | Kose, E & Sezer, RE; 2020 [82] ***** | 316 | 39.6 |  |  |  |  |  |  |
| **38** | 2020 | Istanbul | Kilic, SS & Eren, N; 2022 [83] ***** | 425 | 35.1 |  |  |  |  |  |  |
| **39** | **2020** | **Istanbul** | **Bayramlar, OF, et al.; 2023** | **529** | **32.7** | **3±2.6** | **46.3** | **24.7** | **9.3** | **14.2** | **5.6** |
| **40** | 2020 | Malatya | Bektas, D & Genc, MF; 2020 [84] ***** | 267 | 30.3 |  |  |  |  |  |  |
| **41** | 2020 | Isparta | Aldemir, S & Yigitoglu, G; 2021 [85] ***** | 340 | 23.8 |  |  |  |  |  |  |
| **42** | 2021 | Antalya | Ozdemir M & Ozen, M; 2023 [86] ***** | 165 | 33.3 |  |  |  |  |  |  |
| **43** | 2021 | Ankara | Kamisli, F & Sengezer, T; 2018 [87] ***** | 247 | 40.4 |  | 47.3 | | 30.1 | 22.6 | |
| **44** | 2021 | Izmir | Bolec, B & Birgili, F; 2021 [88] ***** | 203 | 46.3 |  |  |  |  |  |  |
| **45** | 2021 | Ankara | Abbasova, A & Ozturk, C; 2022 [89] ***** | 310 | 51 |  |  |  |  |  |  |
| **46** | 2021 | TÜRKİYE | Polat, S & Ergun, G; 2022 [90] ***** | 253 | 56.5 |  |  |  |  |  |  |
| **47** | 2021 | Mus | Menekse, M & Olca, SP; 2022 [91] ***** | 234 | 57.7 |  |  |  |  |  |  |

*****: Thesis
